# Correlates of loss to follow-up and missed diagnosis among HIV-exposed infants throughout the breastfeeding period in southern Mozambique

**DOI:** 10.1371/journal.pone.0237993

**Published:** 2020-08-21

**Authors:** Maria Grazia Lain, Sergio Chicumbe, Ana Rosa de Araujo, Esmeralda Karajeanes, Aleny Couto, Carlo Giaquinto, Paula Vaz

**Affiliations:** 1 Fundação Ariel Glaser contra o SIDA Pediátrico, Maputo, Mozambique; 2 Health System Program, Instituto Nacional de Saúde, Maputo, Mozambique; 3 HIV Program, Ministry of Health, Maputo, Mozambique; 4 Department for Woman and Child Health, University of Padua, Padua, Italy; University of Ghana College of Health Sciences, GHANA

## Abstract

**Introduction:**

Complete follow-up of human immunodeficiency virus (HIV)-exposed infants (HEI) is crucial for a successful prevention of mother-to-child HIV transmission. This study analyzed the HEI follow-up and factors associated with loss to follow-up (LTFU) in southern Mozambique.

**Methods:**

This retrospective cohort study used the data of HEI enrolled between June 2017 and June 2018, followed-up for 18 months. The outcomes were the proportion of infants with completed follow-up and a definitive diagnosis, and the presence of clinical events. Kaplan–Meier survival analysis was used to calculate the cumulative probability of LTFU and of clinical events. Factors associated with LTFU and clinical events were analyzed using Cox regression to calculate the hazard ratio (HR) and adjusted HR (AHR), with a 95% confidence interval (CI) and a significance cutoff of *p*<0.05.

**Results:**

1413 infants were enrolled (49% males) at a median age of 32 days (IQR 31–41); the median follow-up time was 12 months (IQR 8.2–14.2); 1129 (80%) completed follow-up and had a definitive diagnosis, 58 (4%) were HIV-positive, 225 (16%) were LTFU; 266 (19%) presented a clinical event. Factors associated with LTFU were: age >2 months at entry (AHR, 1.58; 95% CI, 1.12–2.23), non-exclusive breastfeeding (AHR, 1.44; 95% CI, 1.01–2.06), poor cotrimoxazole adherence (AHR, 3.42; 95% CI, 1.59–7.35), and clinical events (AHR, 0.51; 95% CI, 0.34–0.77). Factors associated with clinical events were: malnutrition (AHR, 10.06; 95% CI, 5.92–17.09), non-exclusive breastfeeding (AHR, 1.98; 95% CI, 1.34–2.93), no nevirapine prophylaxis (AHR, 1.67; 95% CI, 1.18–2.36), and poor cotrimoxazole adherence (AHR, 2.62; 95% CI, 1.10–6.22).

**Conclusion:**

The high rate of HEI LTFU, associated with delayed linkage to postnatal care, poor prophylaxis adherence, non-exclusive breastfeeding, indicates the need to design a differentiated service delivery model that is tailored to the mothers’ and infants’ specific needs.

## Introduction

Eliminating the vertical transmission of the human immunodeficiency virus (HIV) requires strong linkages along the prevention of mother-to-child transmission (PMTCT) pathway until infection is ruled out among all HIV-exposed infants (HEI). HEI have increased risk of morbidity and mortality than HIV-unexposed uninfected infants (HUI) [1–6] and deserve special attention until follow-up is completed.

Since the beginning of the HIV epidemic response, guidelines for the care of HEI have been issued [7–9]. In low-income countries, HIV programs have moved from a vertical to an integrated approach to offer care through maternal and child health (MCH) services at the primary level in a strategy to reach all HEI in need and reduce the loss to follow-up (LTFU) [10]. However, early linkage to postnatal care (PNC) to perform the first HIV test and the completeness of follow-up until a definitive diagnosis are not progressing optimally [11–13]. In the priority countries of the Joint United Nations Programme on HIV/AIDS (UNAIDS) Global Plan [14], only 52% of HEI accessed early infant diagnosis (EID) before 6 weeks of age in 2018 [15]. A pooled estimate showed up to 45.5% LTFU before a definitive diagnosis among infants who underwent the first HIV test [11].

In Mozambique, HIV prevalence among women of childbearing age is 15.4% and vertical transmission is estimated at18%, with large variations among the provinces [16,17]. Despite the provision of integrated care at MCH clinics to mother–infant pairs, the rolling out of lifelong antiretroviral therapy (ART) for all pregnant and breastfeeding women living with HIV (Option B+, in 2013), and the achievement of 95% ART coverage [17,18], prevailing challenges still prevent all women and their HEI from receiving prompt and appropriate care. The seroconversion of women during the postpartum and breastfeeding period is estimated to be 4.9 per 100 women-years [19]; moreover, 27% of children who are younger than 2 years have an unknown HIV status [20], and only 66% of expected HEI accessed EID within 2 months of life [18].

Many studies in Sub-Saharan Africa, including in Mozambique, reported data on the retention of HIV-infected pregnant women [21], whereas few studies have described infant follow-up during the entire period at risk of vertical transmission after an initial negative virologic test [11,22]. Moreover, there is poor availability of evidence on factors associated with HEI LTFU, and understanding the correlates of LTFU is key to design better health systems and community response to prevent the dropouts of infants and their mothers from care [23].

In Mozambique, there is a paucity of data on long-term HEI follow-up. This study aimed to describe the completeness of follow-up until definitive diagnosis among HEI, who were enrolled in routine care, the presence of clinical events during follow-up and to analyze factors associated with LTFU and to clinical events.

## Methods

### Setting and study population

This retrospective cohort study included HEI enrolled in the Child at Risk Consultation (CCR) sector of MCH clinics, between June 1, 2017 and June 30, 2018, in four health facilities (HF) located in the Maputo province, in the districts of Boane, Matola, and Marracuene; these facilities have been deliberately de-identified. The follow-up duration was 18 months from enrollment. Data extraction from patients’ medical files was performed from June to September 2019. The HF were specifically selected from among those with high patient volume at the MCH and CCR clinics (average, 30 patients/per MCH nurse/per day) and with the Alere^™^ q HIV-1/2 Detect System–a point-of-care technology for HIV nucleic acid testing diagnosis, using the polymerase chain reaction (PCR) technique, that provides same-day results [24].

### Routine care for HEI and their mothers

The HEI are routinely enrolled in the CCR from 4 weeks of age along with their mothers. At entry, an HIV virologic test (PCR assay) or a serologic test (Rapid test) is offered according to the infant’s age, and a monthly clinical follow-up is done until an HIV definitive diagnosis–HIV-positive or HIV-negative–is established (Table 1).

**Table 1:** The Ministry of Health package of care offered to HEI.

Components of care for HEI	
**HIV virologic test (PCR assay)**	*At entry*, *from 4 weeks to 9 months of age*. At any time in case of any symptoms suggestive of HIV infection *At 9–18 months of age*, *in case of an indeterminate or positive HIV rapid test result*
**HIV serologic test (Rapid test)**	At 9–18 months of age At ≥ 9 months of age and 2 months after breast-feeding cessation
**Cotrimoxazole prophylaxis**	Started at 4 weeks of age and continued until definitive diagnosis
**Nevirapine prophylaxis**	Started at birth and continued until 6 weeks of age
**Clinical evaluation Disease management if needed**	At every monthly visit
**Immunization**	Per national immunization calendar
**Vitamin A supplementation**	Given at 6 months of age and then every 6 months until discharge from CCR
**Deworming**	Given at 12 months of age and then every 6 months until discharge from CCR

An HIV-positive diagnosis is defined as two positive virologic test results at any age, or a positive result on an HIV rapid test at ≥18 months of age. An HIV-negative diagnosis is defined as a negative result on an HIV rapid test performed 2 months after cessation of breastfeeding and when older than 9 months. An indeterminate HIV diagnosis is defined as an indeterminate HIV rapid test result [25,26]. HIV-infected infants are referred to the HIV clinic and start ART, whereas HIV-uninfected infants are referred to the Healthy Child Clinic (HCC). Mothers are followed up with their infants and receive ART, clinical assessment, viral load monitoring, counseling for treatment adherence and retention in care as well as counselling for feeding practices and infant care [27].

### Data collection and analysis

Data were extracted through a retrospective review of the patients’ medical files, filled in by the nurse at each monthly visit. The variables collected for this study were: HF, place and type of delivery, mother on ART at entry, infant’s sex, birth date, NVP prophylaxis at entry, feeding practice at entry, cotrimoxazole at all visits, nutritional classification at all visits, HIV tests (virologic and serologic) performed during follow-up, HIV test results, clinical events during follow-up, transfer to another HF, and death.

The primary outcome was the proportion of HEI with a completed follow-up, defined as an infant with a definitive diagnosis (HIV-positive or HIV-negative) who has been transferred to the HIV clinic or the HCC clinic, respectively. An infant was defined as LTFU when he did not complete all of the follow-up visits to undergo the final test to establish the HIV definitive diagnosis. Infants who had an indeterminate HIV test result reported in the file as the last test result, were also considered to be LTFU, as they did not complete all of the follow-up visits to establish a definitive diagnosis. The LTFU was censored at 18 months of age for each enrolled child.

The secondary outcome was the number of clinical events that occurred during follow up. A clinical event was defined by considering the reported symptoms and the treatment received, and was classified according to the syndromic approach of the Integrated Management of Childhood Illnesses [28] as respiratory infection, gastrointestinal disease, fever of unknown origin, skin problems, malnutrition, and TB exposure.

Descriptive analysis was used to summarize the HEI baseline and follow-up characteristics as well as the mothers’ characteristics at enrollment. The Kaplan–Meier survival function was used to calculate the cumulative probability of LTFU and of clinical events throughout the follow-up. The factors associated with LTFU and clinical events were analyzed using Cox regression to calculate the hazard ratio (HR) and adjusted HR (AHR), with 95% confidence intervals (CI); the following explanatory variables were included: HF, type and place of delivery, mother on ART, infant’s sex, age at entry, birth weight, NVP prophylaxis at entry, feeding practice at entry, cotrimoxazole at all visits, weight-for-height Z-score (WHZ-score) at entry, clinical event during follow-up. We considered significant a correlation with a cut-off level of *p <0*.*05* and with a CI not crossing the value of 1. The adjusted analysis was applied to explanatory variables that showed a significant *p*-value in the unadjusted analysis. All analyses were performed using the Statistical Package for Social Sciences (SPSS) version 23.0 [29].

### Ethical considerations

De-identified data for this study have been retrospectively extracted from patient files. This study was approved by the National Bioethics Committee and Ministry of Health (MOH) (reference IRB00002657 83/CNBS/2017), and the need for informed consent from the patients was waived.

## Results

### Characteristics of HEI and their mothers

A total of 1413 infants (687 male) were enrolled, with a median age of 32 days (IQR 31–41); 1392 (98.5%) had a WHZ-score ≥−1 SD, and 1199 (85%) received exclusive breastfeeding (EBF). At entry, 1159 (82%) were on NVP prophylaxis and 1398 (99%) mothers were on ART (Table 2).

**Table 2:** Characteristics of enrolled HEI and their mothers.

		N (= 1413)	Percent (%)	95% Confidence Interval	
**Health Facility**	HF 1	217	15.4	13.5	17.2
	HF 2	594	42.0	39.5	44.6
	HF 3	290	20.5	18.4	22.6
	HF 4	312	22.1	19.9	24.2
**Gender**	Female	721	51.2	48.4	53.6
	Male	687	48.8	46.0	51.2
	Missing	5			
**Institutional Delivery**	No	48	3.4	2.5	4.3
	Yes	1348	96.6	94.3	96.5
	Missing	17			
**Caesarean Section**	No	1253	89.8	87.0	90.3
	Yes	143	10.2	8.5	11.7
	Missing	17			
**Mother on ART**	No	15	1.1	0.5	1.6
	Yes	1398	98.9	98.4	99.5
**On NVP at entry**	No	254	18.0	16.0	20.0
	Yes	1159	82.0	80.0	84.0
**Feeding practice at entry**	Formula	62	4.4	3.3	5.5
	EBF	1199	85.0	83.0	86.7
	Mixed	97	6.9	5.5	8.2
	Compl. Feeding	53	3.8	2.8	4.7
	Missing	2			
**WHZ-score at entry**	≥-1 SD	1392	98.8	97.9	99.1
	≥-2 <-1 SD	6	0.4	0.1	0.8
	≥-3 <-2 SD	9	0.6	0.2	1.1
	<-3 SD	2	0.1	0.0	0.3
	Missing	4			
**CTX adherence**	No	20	1.4	0.8	2.0
	Yes	1387	98.6	97.5	98.9
	Missing	6			
**Number of children with at least 1 clinical event**	No	1146	81.2	79.1	83.1
	Yes	266	18.8	16.8	20.9
	Missing	1			
**Clinical events**	Respiratory	85	32.0	4.8	7.3
	GI	79	29.7	4.4	6.8
	Fever	20	7.5	0.8	2.0
	Skin Problem	9	3.4	0.2	1.1
	TB exposure	13	4.9	0.4	1.4
	Malnutrition	60	22.6	3.2	5.3
	Total	266	100.0	16.8	20.9
	No clinical event	1147			
**Virologic test (PCR assay) result at entry**	Negative	1337	94.6	93.4	95.8
	Positive	53	3.8	2.8	4.7
	NA*	22		0.9	2.2
	Missing	1			
**Any Positive Virologic (PCR assay) test**	No	1356	96.0	94.9	97.0
	Yes	57	4.0	3.0	5.1
**Indeterminate HIV Rapid test result without a virologic test done**	No	1139	80.6	78.5	82.7
	Yes	274	19.4	17.3	21.5
**Outcomes of follow-up**	LTFU	143	10.1	8.5	11.7
	HIV-indeterminate	82	5.8	4.6	7.0
	HIV-negative (HCC)	1071	75.8	73.6	78.0
	HIV-positive (HIV clinic)	58^§^	4.1	3.1	5.1
	Death	8	0.6	0.2	1.0
	Transferred	51	3.6	2.6	4.6

Abbreviations: HF = Health facility; ART = antiretroviral therapy; NVP = nevirapine; CTX = cotrimoxazole; Compl. = complementary; EBF = exclusive breastfeeding; GI = gastro-intestinal; TB = tuberculosis; PCR = polymerase chain reaction; HCC = Healthy Child Clinic;

*NA = not applicable, includes infants older than 9 months of age at entry, who are not eligible to receive a virologic test as first HIV test, but they receive a Rapid test;

^§^1 child underwent the Rapid test at entry at 18 months of age.

### HEI follow-up and HIV diagnosis

**Fig 1:**
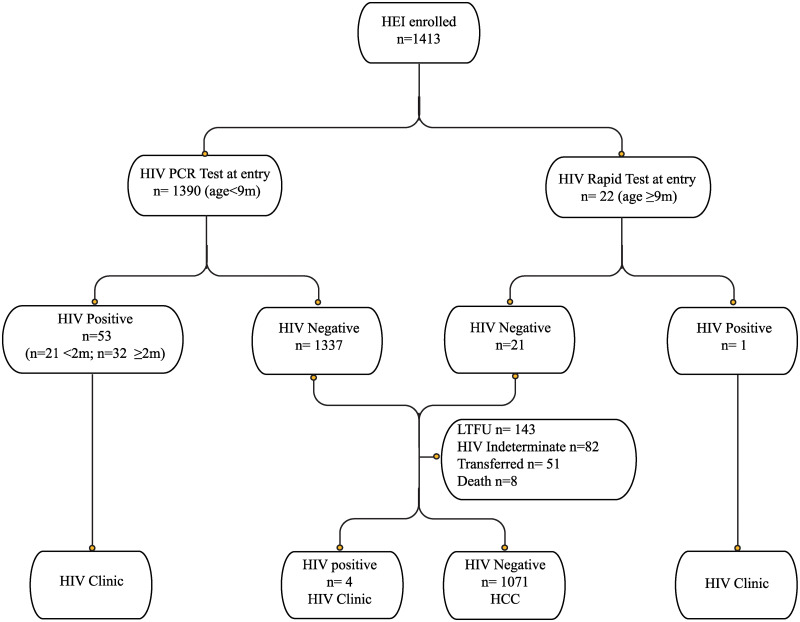
HEI follow-up cascade.

The median follow-up time from enrollment was of 12 months (IQR 8.2–14.2) and the median age at the last visit was of 13 months (IQR 11–16), respectively. A total of 1129 (80%) infants completed follow-up and had a definitive diagnosis (Fig 1).

A total of 58 (4%) infants were diagnosed as HIV-positive: 54 (3.8%) at the first visit and 4 during follow-up visits; 32 (56%) of these infants were older than 2 months at HIV diagnosis. All HIV-positive infants were referred to the HIV clinic to start ART. A total of 1071 (75.8%) infants were diagnosed as HIV-negative and were referred to the HCC. Moreover, 274 (19.4%) infants whose HIV rapid test result at 9 months was indeterminate did not receive a virologic test on the same day: 136 of those repeated a rapid test 3 months later, 3 had an HIV-positive result, and 82 did not repeat any HIV test and had no definitive diagnosis.

A total of 225 (16%) infants were LTFU: 46% and 65% dropped out within the first 3 and 6 months after enrolment, respectively. The cumulative probability of LTFU at 3, 6, and 9 months was of 8%, 11%, and 13.5% respectively (S1 Fig).

Fifty-one infants (3.6%), with a first negative virologic test, were transferred to another HF before a definitive diagnosis was established: 71% were transferred within the first 3 months after enrolment and 96% within the first 6 months. Eight (0.6%) infants died before completing follow-up.

### Factors associated with HEI LTFU

In the multivariate analysis, factors that were associated with LTFU were: age at entry >2 months (AHR, 1.58; 95% CI, 1.12–2.23), non-EBF at entry (AHR, 1.44; 95% CI, 1.01–2.06), poor adherence to cotrimoxazole prophylaxis during follow-up (AHR, 3.42; 95% CI, 1.59–7.35), and the presence of a clinical event during follow-up (AHR, 0.51; 95% CI, 0.34–0.77) (Table 3).

**Table 3:** Factors associated with HEI LTFU.

	Bivariate Analysis					Multivariate Analysis				
	Wald Chi^2^ Test	*p value*	HR	95% CI		Wald Chi^2^ Test	*p value*	AHR	95% CI	
				Lower	Upper				Lower	Upper
HF 1*	5.34	0.149								
**HF 2**	1.97	0.161	1.36	0.88	2.10	-	-	-	-	-
**HF 3**	1.48	0.223	1.25	0.87	1.79	-	-	-	-	-
**HF 4**	0.35	0.557	0.87	0.56	1.37	-	-	-	-	-
Non-institutional delivery*	0.31	0.577	1.21	0.62	2.36	-	-	-	-	-
Caesarean section*	0.07	0.790	1.06	0.69	1.63	-	-	-	-	-
Male*	0.01	0.905	0.98	0.76	1.28	-	-	-	-	-
Birth Weight*	0.03	0.861	0.98	0.73	1.29	-	-	-	-	-
Mother Not on ART*	0.36	0.549	1.53	0.38	6.17	-	-	-	-	-
**No NVP prophylaxis**	1.48	0.224	1.23	0.88	1.73	-	-	-	-	-
**Age > 2m at entry**	11.43	0.001	1.77	1.27	2.45	6.79	0.009	1.58	1.12	2.23
**Non-EBF at entry**	10.87	0.001	1.75	1.26	2.45	3.97	0.046	1.44	1.01	2.06
**Poor adherence to CTX**	10.61	0.001	3.51	1.65	7.46	9.88	0.002	3.42	1.59	7.35
WHZ-score < -1SD at entry*	0.00	0.947	1.05	0.26	4.22	-	-	-	-	-
**Clinical event**	9.49	0.002	0.53	0.36	0.79	10.27	0.001	0.51	0.34	0.77

*Variable not analyzed in the Multivariate analysis because its association was not significative in the Bivariate analysis.

Abbreviations: HF = Health facility; ART = antiretroviral therapy; NVP = nevirapine; EBF = exclusive breastfeeding; CTX = cotrimoxazole; WHZ = weight-for-height Z-score.

### Clinical events during follow-up

A total 266 infants presented with at least one clinical event during follow-up: 32% had respiratory tract infections, 30% had gastrointestinal disease, 23% had malnutrition, 7% had fever of unknown origin, and 3% had skin problems (Table 2).

The factors associated with clinical events were: malnutrition at entry (AHR, 10.06; 95% CI, 5.92–17.09), non-EBF (AHR, 1.98; 95% CI, 1.34–2.93), no NVP prophylaxis (AHR, 1.67; 95% CI, 1.18–2.36), and poor adherence to cotrimoxazole prophylaxis during follow-up (AHR, 2.62; 95% CI, 1.10–6.22) (Table 4).

**Table 4:** Factors associated with the presence of a clinical event during follow-up.

	Bivariate Analysis					Multivariate Analysis				
	Wald Chi^2^ test	p value	HR	95% CI		Wald Chi^2^ test	p value	AHR	95% CI	
				Lower	Upper				Lower	Upper
**HF 1**	102.36	0.000				114.16	0.000			
**HF 2**	0.01	0.915	1.02	0.73	1.42	0.02	0.883	1.03	0.72	1.46
**HF 3**	78.14	0.000	0.26	0.19	0.35	91.02	0.000	0.22	0.16	0.30
**HF 4**	27.27	0.000	0.34	0.23	0.51	25.57	0.000	0.35	0.23	0.52
Non-institutional Delivery*	0.31	0.578	1.18	0.66	2.11	-	-	-	-	-
Caesarean section*	0.00	0.955	0.99	0.64	1.52	-	-	-	-	-
Male*	1.51	0.219	0.86	0.67	1.09	-	-	-	-	-
Birth weight*	0.01	0.913	1.01	0.78	1.32	-	-	-	-	-
**Mother not on ART**	20.85	0.000	6.66	2.95	15.02	2.67	0.102	2.21	0.85	5.73
**No NVP prophylaxis**	12.21	0.000	1.71	1.26	2.30	8.27	0.004	1.67	1.18	2.36
**Age >2m at entry**	12.95	0.000	1.88	1.33	2.64	0.07	0.786	1.06	0.70	1.60
**Non-EBF at entry**	8.07	0.004	1.67	1.17	2.38	11.85	0.001	1.98	1.34	2.93
**Poor adherence to CTX**	21.57	0.000	6.00	2.82	12.77	4.77	0.029	2.62	1.10	6.22
WHZ-score < -1SD at entry*	60.61	0.000	7.68	4.60	12.82	72.98	0.000	10.06	5.92	17.09

*Variable not analyzed in the Multivariate analysis because its association was not significative in the Bivariate analysis.

Abbreviations: HF = Health facility; ART = antiretroviral therapy; NVP = nevirapine; EBF = exclusive breastfeeding; CTX = cotrimoxazole; WHZ = weight-for-height Z-score.

The cumulative probability of a clinical event at 3, 6, 9, and 12 months was 1.6%, 2.5%, 6%, and 19% respectively (S2 Fig).

## Discussion

Our analysis showed that a high proportion (16%) of HEI did not complete follow-up and did not have a definitive diagnosis. This novel evidence highlights a critical gap in the postnatal continuum of the PMTCT cascade in Mozambique. In our cohort, approximately half of the LTFU infants dropped out within the first 3 months after enrolment (median age, 4 months), at an age that is associated with the highest risk of morbidity and mortality [30]. Moreover, infants continue to be at risk of HIV transmission during the breastfeeding period, especially if the mother stops ART [31,32]. Similarly, a study in Kenya reported that 43% of drop-outs occurred within 2 months of enrolment in the PNC [33]. In contrast, a recent study reported that the majority of HEI were LFTU between 9 and 18 months of age [22]. Knowing the potential timepoint when the mother–infant pair is more likely to leave care is important to strengthen effective retention interventions in the period when drop-outs are more likely to occur.

Several causes may determine drop-out from care, including clinical, psychosocial, and health system structural factors [39–42]. In our cohort, HEI accessing care at age ≥2 months presented a higher risk of LTFU than those who were enrolled at less than 2 months of age. The delayed enrolment may be due to the lack of access of the women to the ANC [34,35] and because of missed counselling sessions on the importance of early PNC linkage for their infants. However, our data showed that almost all mothers were on ART at the time of enrolment and had institutional deliveries, which suggests previous contact with health staff although eventually receiving inadequate counseling or being unable to link to the program early, within 2 months after the baby was born.

In fact, at high-volume MCH clinics, nurses or counselors may be unable to spend sufficient time for an appropriate counseling session, as shown in a study in central Mozambique where the median duration of an infant’s visit was between 5 and 11 minutes and the feedback from interviews with mothers and health workers suggested a need to improve the counseling sessions [36].

Although access to EID in Mozambique improved from 36% in 2010 to 66% in 2018 [17] a large proportion of infants were not linked to the PNC within 2 months of age. In Zambezia, in central Mozambique, enhanced referral from maternity clinics to the PNC has been shown to increase linkage to EID three-fold [37]. In neighboring countries such as South Africa and Eswatini, HEI linkage to PNC for EID within the first 2 months after delivery was 80% and 78%, respectively [38]. Therefore, additional effort is required to decrease the LTFU rate and improve early access to PNC, which allows HEI to undergo the HIV test and benefit from the enhanced postnatal HIV prophylaxis that was recently recommended by the World Health Organization (WHO) and adopted by the MOH [26].

This study did not evaluate socioeconomic determinants, such as lack of partner or family support, fear of stigma among others. However, these factors might have affected the early access to PNC and retention throughout the cascade, as reported by other studies conducted in Mozambique, South Africa, and Malawi [36,39–41]. Therefore, collecting such data in future studies is crucial to understand whether early linkage and completeness of follow-up are influenced by the same determinants that affect access and retention along all of the steps of the PMTCT cascade [20,40,42].

We found a higher risk of HEI LTFU related to non-EBF at entry, which is similar to the result of a study conducted in Uganda wherein early weaning was associated with a high risk of LTFU [43]. In Mozambique, EBF during the first 6 months of life is the norm, and women are counseled on the best feeding practices at all ANC and PNC visits [27]. Our data suggest the possibility that either the mothers did not access ANC or they did not receive proper feeding counseling. Again, the quality of counseling sessions is crucial to convey appropriate healthcare messages, and it is critical to keep infants linked to care [36].

Poor adherence to cotrimoxazole prophylaxis was another risk factor for LTFU, and it has been already shown to be associated with poor adherence to care [44]. It is important to emphasize that several predictors of HEI LTFU also correlate with HIV VT [45]. Thus, MCH nurses, counselors, and mentor mothers should provide special attention to the first ANC visit of women presenting risk factors for poor adherence to ART and retention in care, and thereby intensify support.

In our clinical setting, PMTCT care is integrated into MCH services, as recommended by the WHO [8]. However, our findings suggest that health services integration alone, which has proven successful in Mozambique to increase HIV testing and treatment coverage among pregnant and breastfeeding women [17], is inadequate to address the postnatal linkage gap and to ensure completeness of HEI care. Our suggestion is aligned with a recent review, which included many African studies, that described weak evidence of improved mother–infant pair retention due to the integration of PMTCT interventions into MCH care [46].

Synergism between health facility and community-based interventions is key to complement the achievements obtained through the reorganization of services delivery at the HF level. Text messages to mothers during the postnatal period [47,48], partner’s involvement in PMTCT care and group counseling sessions [49–51], mentor mothers’ strategy [52,53], and facility and community-based peer support [54] resulted in an increased 12–24 month mother–infant pair retention rate. Only one study on the efficacy of mother support groups showed no difference in HEI retention at 12 months [55].

Similarly as in another study in Uganda [43], presenting with a clinical event during follow-up decreased the likelihood of LTFU. This reflects the tendency of mothers to seek and stay in care when their baby is sick [41] and suggests the increased attention of counselors to convey messages on the importance of continuing care.

We reported a large number of HEI (n = 274) who did not receive a virologic test at 9 months of age, following an indeterminate rapid test result; eventually, 6% did not complete the follow-up visits and had no definitive diagnosis. This finding highlights some challenges among MCH nurses in applying the EID algorithm correctly at all the steps of the HIV diagnosis cascade, and this contributed to the increased number of HEI without a definitive diagnosis in our cohort. Supportive mentorship to nurses and simplification of diagnosis guidelines may be needed to minimize delays in HIV diagnosis, as suggested by the WHO [25].

Another key finding is that less than half of HIV-infected infants were diagnosed before 2 months of age, which is the recommended age to start ART and to limit morbidity and mortality in this group [30]. Delayed identification of infected infants is mainly due to late PNC enrolment, as discussed above, but is also because of poor active case findings at services with the highest HIV diagnostic yield, such as the pediatric ward and malnutrition clinic [56]. In Mozambique, despite the provider-initiated counseling and testing (PICT) approach that has been adopted since many years [57], a recent study reported that only 46% of children admitted to 11 pediatric wards and eligible for testing received an HIV test [58]. In Zambia and Malawi, the PICT approach at the Under-5 clinics and pediatric wards increased the identification of HIV-positive infants whose mothers did not link to PNC [59–61].

We found that 4% of enrolled infants were transferred to another HF before completing follow-up. To our knowledge, no data on this matter were available so far in Mozambique. Although our data are limited to describe the reasons for being transferred, it is reasonable to consider that the transfer was possibly due to the migration of the family to another location [39] or to the local cultural custom wherein a pregnant women spends the last month of pregnancy and the first months after delivery at the mother’s or relatives’ house [62].

Our evidence highlights the need to address cultural elements, specifically pertaining to pregnant and breastfeeding women, to ensure the PMTCT continuum of care by designing a specific differentiated service delivery (DSD) model for them. In Mozambique, the implementation of DSD models has been scaled-up in 2019 [63,64], but this poorly considered pregnant and breastfeeding women, who should be targeted for proper follow-up.

Clinical events were frequent in our cohort, with the majority being respiratory and gastrointestinal illnesses, as other studies in the literature have reported [1,65,66]. We found that non-EBF at entry and poor cotrimoxazole adherence during follow-up were factors associated with a higher risk of clinical event, confirming that cotrimoxazole and breastfeeding are protective factors against increased morbidity in infants [5,9,67,68]. The young age (4–6 months) at LTFU that we reported in our cohort as well as the higher morbidity and the increased infection severity reported in this age group than in HIV-unexposed infants [1,5,68–73] call for an HIV program to intensify retention interventions and the development of a tool to track the mother–child pair until a definitive diagnosis is established.

We report a vertical transmission rate of 4%, which is lower than the 18% estimated by the Spectrum model–a software used by the national HIV program to prepare estimates of key HIV indicators based on country-specific survey data and evidence from scientific studies [74]. The parameters used in the model to estimate the MTCT rate in Mozambique may need context-specific reconsideration because a similar vertical transmission rate of 5% has been reported at other HFs in the Maputo province [17] and in an HIV prevalence study conducted in the Manhiça district among HEI who were younger than 4 years [75]. However, considering the significant proportion of infants who did not complete follow-up, the vertical transmission rate we described cannot be considered definitive.

This study had a few limitations. In southern Mozambique, health service coverage remains suboptimal, which may have induced recruitment bias. Moreover, due to the retrospective nature of the study and the format of the MOH patient file, we could not collect data on the mother’s socioeconomic determinants, viral load, or CD4 count test results, which are additional factors that can be associated with an increased risk of infant’s LTFU and morbidity [69,76]. A reporting bias may have occurred in completing the patient’s file during the clinical visit. Another limitation is that the results are from HF located in the Maputo province and, therefore, cannot be generalized to other provinces in Mozambique.

Nonetheless, we expect that the factors we found to be related to HEI LTFU are common among pregnant and breastfeeding women living with HIV in Mozambique, and we believe that our findings may be considered a basis to strengthen the PMTCT program in the other provinces as well. Additionally, our results call for the HIV program to conduct a study of the completeness of the PMTCT cascade and factors related to LFTU, even in the other provinces, where the vertical transmission rate is higher than that in Maputo.

A strength of this study is that it was conducted in the “real world” setting of very busy MCH clinics, and the results are more likely to reflect the real outcomes of service delivered to HEI and their mothers.

## Conclusions

The high rate of HEI LTFU before attaining an HIV definitive diagnosis was associated with delayed linkage to postnatal care, poor prophylaxis adherence, non-EBF; all of which jeopardize the elimination of pediatric HIV as well as the success of the pediatric HIV program.

An innovative DSD model that addresses pregnant and breastfeeding women and their infant’s needs should be designed to ensure the infant’s early access to PNC and guarantee long-term retention until HIV infection is ruled out. Counseling and retention activities in the first few months of the infant’s life after PNC enrolment should be intensified while combining existing successful interventions at HF with those in the community.

## Supporting information

S1 Fig Cumulative probability of LTFU of HEI. (DOCX)

S2 Fig Cumulative probability of clinical event during follow-up. (DOCX)
