# Supplementary figures and images for: Correlates of loss to follow-up and missed diagnosis among HIV-exposed infants throughout the breastfeeding period in southern Mozambique
Source: PLoS One. 2020 Aug 21;15(8):e0237993. doi: 10.1371/journal.pone.0237993 (PMC7444585; doi:10.1371/journal.pone.0237993)

**S 1 Figure Cumulative probability of lost to follow-up of HEI**


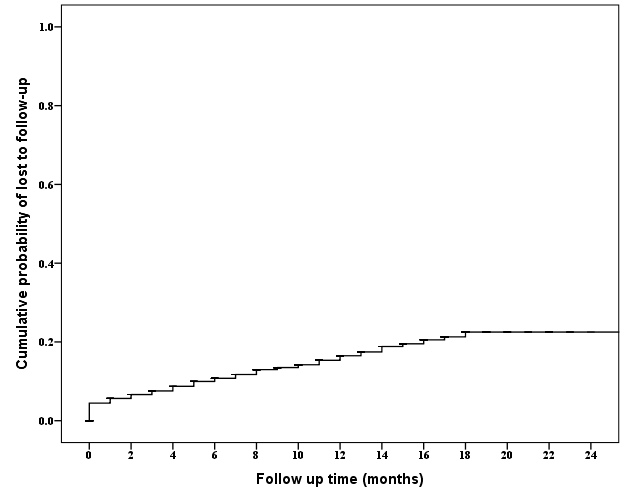

Supplement: S1 Fig — (DOCX) [file pone.0237993.s001.docx]

**S2 Figure. Cumulative probability of clinical event during follow-up**


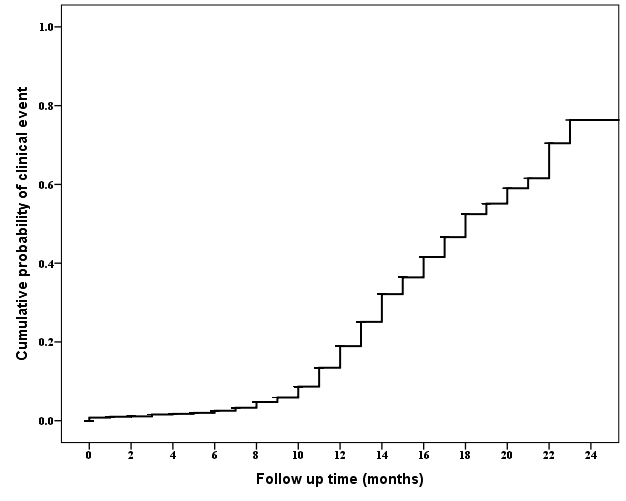

Supplement: S2 Fig — (DOCX) [file pone.0237993.s002.docx]
